# Cognitive impairment and brain atrophy in patients with newly diagnosed aggressive lymphoma undergoing standard chemotherapy: a normative analysis

**DOI:** 10.3389/fneur.2025.1601459

**Published:** 2025-09-17

**Authors:** Charlotte Ditchfield, Priscilla Gates, Juan F Domínguez D, Haryana M. Dhillon, Vincent Dore, Carlene Wilson, Karen Caeyenberghs

**Affiliations:** ^1^School of Psychology, Deakin University, Burwood, VIC, Australia; ^2^Centre for Health Service Research, Peter MacCallum Cancer Centre, Melbourne, VIC, Australia; ^3^Clinical Haematology, Austin Health, Heidelberg, VIC, Australia; ^4^Psycho-Oncology Cooperative Research Group, The University of Sydney, Camperdown, NSW, Australia; ^5^The Australian e-Health Research Centre, CSIRO Health and Biosecurity, Melbourne, VIC, Australia; ^6^Faculty of Medicine, Dentistry and Health Sciences, University of Melbourne, Parkville, VIC, Australia

**Keywords:** cancer-related cognitive impairment, brain atrophy, aggressive lymphoma, normative analysis, chemotherapy

## Abstract

**Objective:**

Cancer-related cognitive impairment (CRCI) can impact daily-life functioning of people with aggressive lymphoma. While many studies have examined the neural substrates implicated in CRCI, most have used group-based analyses, which may mask individual differences. In the present study, we used normative analysis to examine longitudinal changes in cognitive functioning and brain morphology at the level of the individual patient.

**Methods:**

Nine participants with newly diagnosed aggressive lymphoma underwent neuropsychological assessment and anatomical MR before and 6–8 weeks after chemotherapy. Cognitive test scores were converted to *T-*scores and classified as impaired if ≤ 30. Deviations in cortical thickness and surface area in the superior frontal gyrus (SFG) and anterior cingulate cortex (ACC) were computed at the level of the individual using the novel CentileBrain tool, with *z*-scores below − 1.96 and above 1.96 classified as infranormal and supranormal, respectively.

**Results:**

Analyses revealed substantial between-subject variability over time and across outcome measures. Cognitive impairments in executive function and verbal memory were identified in three participants before and/or after chemotherapy. CentileBrain results showed seven participants had infranormal cortical thickness and/or surface area in the SFG at one or both time points, and one patient had infranormal values in the ACC. No participants exhibited supranormal values in either region at any time point.

**Conclusion:**

Our findings provide a valuable foundation for developing more personalised interventions tailored to the specific cognitive and neural profiles of lymphoma survivors, paving the way for improved clinical care to alleviate and mitigate the impact of CRCI on long-term quality of life.

**Clinical trial registration:**

https://www.anzctr.org.au/, identifier ACTRN12619001649101.

## Introduction

Aggressive lymphoma is a type of cancer caused by the rapid proliferation of malignant lymphocytes, categorised as Hodgkin lymphoma (HL) and non-Hodgkin lymphoma (NHL). The most common is diffuse large B-cell lymphoma (DLBCL), with an estimated incidence of 2000 Australians annually (1). Advances in diagnosis and treatments have improved survival rates (2), with 5-year survival rates ranging between 74 and 95% (3, 4), and stable remissions in 68–88% of individuals (5). However, this has led to a growing population of survivors living with the physical and neuropsychological sequelae of cancer and its treatment.

Many studies have demonstrated people with aggressive non-central nervous system (non-CNS) lymphoma experience poorer physical and emotional wellbeing, compared with the general population (6, 7). Additionally, studies have shown these people experience cancer-related cognitive impairment (CRCI) (6, 8), a term used to describe impaired functioning in domains including memory, attention, executive functioning, and processing speed that may arise before, during, or after cancer treatment as a consequence of the disease and its therapies (9). While prevalence varies across studies, research indicates 13–70% of cancer survivors experience cognitive dysfunction during or after treatment (10). These symptoms can last for years with a profound impact on cancer survivors’ quality of life, occupational and social functioning, and daily activities (9, 11, 12).

CRCI has been measured through subjective self-report and objective neuropsychological assessments, evaluating various cognitive functions including executive functioning, memory, attention, and processing speed (13, 14). Although little research has been conducted in people with aggressive lymphoma, a multitude of studies have been conducted in breast cancer and other non-CNS cancer groups, establishing evidence of CRCI across a wide array of cognitive domains (9, 14, 15). Some literature has included people with haematological malignancies (6, 8, 16–18). Cross-sectional studies have demonstrated aggressive lymphoma survivors have significantly lower subjective and/or objective cognitive functioning compared to healthy controls or normative data, even years after the completion of chemotherapy (17, 18). Recently, two longitudinal studies have been conducted, helping to disentangle the effects of cancer and chemotherapy over time (6, 8). For example, Janelsins et al. (8), observed significant cognitive decline in tests of memory, attention and executive function in people with aggressive lymphoma from before to six months after chemotherapy, compared to healthy controls. Similarly, Gates et al. (6) found that compared to healthy controls, people with aggressive lymphoma performed significantly worse, both before and 6 to 8 weeks after chemotherapy, on measures of processing speed, executive function, learning and memory. Participants also reported their perceived cognitive impairment had a significantly greater impact on their quality of life compared to controls, and this difference was evident both before and after chemotherapy. This highlights the impact of CRCI and its effect on the quality of life for people with aggressive lymphoma.

Several magnetic resonance imaging (MRI) studies have attempted to elucidate the neural underpinnings of CRCI. Data have consistently shown reductions in grey matter density, alongside alterations in white matter microstructure and brain activation and connectivity in cancer survivors (14, 19–24). Most of these MRI studies have included people with breast cancer, employing a cross-sectional or retrospective design (25–27). However, several longitudinal studies including other or mixed non-CNS cancer populations have demonstrated similar findings (14, 28–31). For example, a systematic review of 14 longitudinal studies identified moderate changes in grey matter density in frontal, parietal and temporal brain regions in non-CNS cancer populations after chemotherapy (14). While some structural differences at baseline have been observed that could be attributed to the disease process, these changes tend to become more pronounced following chemotherapy (19). Although the biological mechanisms are unclear, the neurotoxic side effects of chemotherapy – such as DNA damage, oxidative stress, hormonal dysregulation and inflammation – are thought to contribute to neuroanatomical alterations (32). Indeed, longitudinal studies with a control group of people with cancer not treated with chemotherapy have shown some neuroanatomical changes are directly linked to chemotherapy rather than the cancer itself (19, 33–35). For instance, anatomical MRI studies found breast cancer survivors had reduced grey matter density, particularly in frontal regions, one month after chemotherapy, while no significant changes were evident in people not treated with chemotherapy (19, 34).

Although the presence of grey matter density reductions has been consistently observed in frontal regions, the exact subregions in this lobe have varied across studies (14). To provide a more precise understanding of these variations in anatomical location, a recent voxel-wise meta-analysis of eight studies revealed cancer survivors treated with chemotherapy exhibited significantly reduced grey matter density in the right superior frontal gyrus (SFG) and right anterior cingulate (ACC) compared to non-cancer controls and cancer survivors not treated with chemotherapy (24). Interestingly, reduced grey matter volume in these regions has been shown to correlate with objective and subjective cognitive functioning (14, 19, 26). For example, Inagaki et al. found that, in cancer survivors one year after chemotherapy, greater atrophy in the right SFG was associated with poorer visual memory and attention/concentration, while atrophy in the cingulate gyrus was associated with poorer working memory (26). Despite these findings, only two studies, to our knowledge, have examined alterations in brain volume specifically in people with haematological malignancies (29, 30). These studies identified neuroanatomical changes from baseline to one-year post-haematological stem cell transplantation (HSCT), which involves high-dose chemotherapy. People exhibited decreased grey matter volume in the bilateral middle frontal gyrus and left caudate nucleus (30), and reduced mean and axial diffusivity in diffuse brain regions (29). However, these studies lacked a true baseline because they had previously received chemotherapy unrelated to the HSCT regimen.

Although previous studies have advanced knowledge on grey matter volume changes in cancer populations, much less is known about the specific roles of cortical thickness and surface area as distinct measures of brain atrophy (36–38). Cortical thickness and surface area represent different aspects of cortical structure: thickness is linked to the density of cells within cortical columns, while surface area relates to the number of these columns in a given region (39). These two measures are shaped by different cellular processes due to their unique genetic and environmental determinants, suggesting that the factors influencing the variation in cortical thickness may differ from those affecting the variation in surface area (39, 40). Furthermore, because grey matter volume is a product of both thickness and surface area, disentangling these components can provide more biologically specific insights into patterns of brain atrophy (41). However, research examining cortical thickness and surface area changes in cancer populations is limited, with only three known studies using these metrics (37, 38, 42). Wu et al. (38) found, compared to healthy controls, people with lung cancer had significantly lower pre-chemotherapy cortical thickness in several regions, and it was the only measure that declined further after chemotherapy, whereas volume changes were restricted to the temporal regions and no changes were observed in surface area. Conversely, Mentzelopoulos et al. (37) identified reductions in both cortical thickness and volume across multiple brain regions after chemotherapy, while Shiroishi et al. (42) observed decreases in either cortical thickness or surface area in various regions. This highlights the importance of examining cortical thickness and surface area separately, as each measure may capture unique aspects of brain atrophy that could be overlooked when focusing solely on volume changes.

While the above-mentioned findings have provided valuable insights into cognitive and neuroanatomical changes in cancer populations, they are based on group-wise comparisons (i.e., patient vs. control groups) which may obscure important individual differences (20). Traditional group analyses cannot adequately reflect what happens in individual patients or adequately handle between-patient heterogeneity. Moreover, clinicians need to perform diagnostic and prognostic inferences at the level of individual patients. It is important to investigate these changes using normative analysis in order to take into account the inherent heterogeneity within these cancer populations. It is not surprising that cognitive and neuroimaging findings have been mixed, considering the variability in demographic and clinical characteristics of cancer survivors, including variables such as cancer type, disease stage, treatment regime, and number of cycles of chemotherapy (23). This variability has been reflected in the heterogeneity of affected cognitive domains and brain regions observed across studies (14), along with findings showing that only a subset of people exhibit cognitive decline following chemotherapy (43–46). Therefore, normative analysis has the potential to reveal subtle neuroanatomical changes not detected in group studies. Specifically, normative modelling can be used to index how much each subject deviates from the normative range from the reference population (via a deviation or a *z*-score) (47, 48). This normative modelling framework explicitly accommodates interindividual variability by treating abnormality as a deviation from an established healthy range rather than a group difference. Specifically, normative modelling uses data from a large control cohort to learn a normative distribution that characterises the full cohort. Normative modelling essentially aims to predict centiles of variance in a response variable (e.g., a region of interest or other neuroimaging-derived measure) on the basis of a set of covariates (e.g., age, gender, etc). While there is a paucity of normative analysis in cancer populations, there is emerging literature using normative analyses in psychiatric (49, 50), and neurological disorders (51, 52). For example, Allen et al. (50) used a novel open-source tool called CentileBrain, which provides a normative framework for regional brain morphometrics, developed from a sample of 37,407 healthy individuals by the ENIGMA Lifespan Group (49, 53). Using CentileBrain, they found mostly overlapping deviations in brain structure between individuals at high risk for psychosis and typically developing groups, but distinct differences in those who later developed a psychotic disorder suggesting potential early markers of conversion to psychosis (54). The present study will perform a normative analysis using CentileBrain to identify individual deviations from typical brain structure in people with aggressive lymphoma. Moreover, we will examine changes in cognitive functioning at the level of the individual patient.

The aims of our study are twofold. Firstly, we will examine changes in cognitive function using a comprehensive set of objective neuropsychological tests recommended by the International Cognition and Cancer Task force (ICCTF) (10), in people with aggressive non-CNS lymphoma before and approximately 6–8 weeks after chemotherapy compared to population norms. We hypothesise that cognitive test scores from individual participants will deviate from the population norms at both timepoints (6), with large between-subject variability in the affected domains. Secondly, using the CentileBrain framework, we will explore alterations in cortical thickness and surface area in these participants from before and 6–8 weeks after chemotherapy. We expect the cortical thickness and surface area values from frontal regions of individual participants, including the SFG and ACC (24), will deviate from the normative group at both timepoints.

## Methods

### Study design

This study presents analyses of the MRI scans from a subsample of a larger-scale longitudinal study cognitive impacts of chemotherapy in people with aggressive lymphoma (55, 56). All participants provided written informed consent. Ethical approval was granted by the Human Research Ethics Committees of Austin Health (HREC 55582/Austin-2019) and Deakin University (DUHREC 2024–024).

### Participants

We included nine participants (7 male, 2 female) aged 29–78 years (*M* = 60.00, *SD* = 14.75) recruited from a specialised haematology department at Austin Health in Melbourne, Australia. Participants had to meet the following inclusion criteria: (i) aged over 18 years; (ii) newly diagnosed with HL, DLBCL, Burkitt lymphoma, transformed follicular lymphoma, or grade 3B follicular lymphoma; (iii) treatment naïve at time of enrolment and scheduled to undergo standard cancer treatment; (iv) fluent in English; and (v) a documented Eastern Cooperative Oncology Group (ECOG) performance status ≤ 2. Exclusion criteria included the following: (i) lymphomatous central nervous system involvement; (ii) prior or planned cranial radiotherapy; (iii) a life expectancy < 12 months; (iv) any medical condition that could compromise adherence or lead to prolonged hospitalisation; (v) a documented history of past or current substance abuse; or (vi) major psychiatric disorder (e.g., schizophrenia). Table 1 provides a summary of the clinical and demographic characteristics of participants.

**Table 1:** Summary of demographic and clinical characteristics.

Participant ID	Age in years	Sex	Years of formal education	Diagnosis	Stage	Chemotherapy regime and number of cycles
Participant 1	47	Male	16	HL	2A	ABVD x 6
Participant 2	66	Male	14	Grade 3B follicular	1A	R-CHOP x 6
Participant 3	69	Male	12	DLBCL	1A	R-CHOP x 7 and high dose IT MTX x 2
Participant 4	78	Female	11	DLBCL	4A	Mini R-CHOP x 6
Participant 5	72	Male	18	DLBCL	1A	R-CHOP x 2
Participant 6	29	Male	14	HL	4B	Esc-BEACOPP x 4
Participant 7	60	Male	11	DLBCL	2A	R-CHOP x 4
Participant 8	63	Female	13	DLBCL	1A	R-CHOP x 3
Participant 9	56	Male	13	DLBCL	2B	R-CHOP x 3

HL, Hodgkin lymphoma; DLBCL, diffuse large B cell lymphoma; ABVD, adriamycin, bleomycin, vinblastine, and dacarbazine; R-CHOP, rituximab, cyclophosphamide, doxorubicin, vincristine, and prednisolone; IT MTX, intrathecal methotrexate; Esc-BEACOPP, bleomycin, etoposide, doxorubicin, cyclophosphamide, vincristine, procarbazine, and prednisolone.

### Procedure

Participants underwent MRI scans and neuropsychological assessments at two time points: (i) pre-chemotherapy at the time of diagnosis; and (ii) approximately 6–8 weeks post-chemotherapy completion. The pre-chemotherapy MRI scans were performed on average 12.9 days (SD = 9.8, range 3 to 29 days) from date of cancer diagnosis. The pre-chemotherapy neuropsychological assessments were performed on average 13 days (SD = 10.9, range 0 to 29 days) from date of diagnosis. The post-chemotherapy MRI scans were performed on average 7.8 weeks (SD = 4.7, range 3 to 15 weeks) from date of chemotherapy completion. The post-chemotherapy neuropsychological assessments were performed on average 9 weeks (SD = 7.3, range 3 to 26 weeks) from date of chemotherapy completion, which is in line with standard clinical practice.

### Neuropsychological assessment

A series of neuropsychological tests were used to assess cognitive functioning in line with recommendations by the ICCTF (10). The cognitive domains included: (i) attention/working memory tested via the Digit Span of the Weschler Adult Intelligence Scale-Revised (WAIS-R) (57), (ii) information processing speed via the Trail Making Test (TMT) Part A (58), (iii) executive function via the TMT Part B (58), and the Stroop Colour and Word Test (SCWT) (59), (iv) verbal learning and memory via the Hopkins Verbal Learning Test-Revised (HVLT-R) (60), and (v) verbal fluency via the Controlled Oral Word Association Test (COWAT) (61). See Table 2 for a detailed overview of the neuropsychological tests.

**Table 2:** Overview of neuropsychological test battery.

Test	Cognitive domain	Instruction for participant	Dependent variable(s)
WAIS-R DS (57)	Attention/ working memory	Recall visually presented numbers of increasing length in the same or reverse order.	Total correct responses
TMT-A (58)	Information processing speed	Connect 25 numbered circles in ascending order quickly and accurately.	Time to complete (seconds)
TMT-B (58)	Executive function	Connect 24 alternating numbers and letters in alternating ascending order quickly and accurately.	Time to complete (seconds)
SCWT (59)	Executive function	Read colour words in black ink, colours of patches and the ink colour of incongruent words.	Time (seconds) for each condition and interference score
HVLT-R (60)	Verbal learning and memory	Recall 12 verbally presented words over three trials, followed by a delayed recall and a 24-word recognition task to identify target words from distractors.	Total and delayed recall, percentage retained and recognition/ discrimination
COWAT (61)	Verbal fluency	Generate words, orally or in writing, from a given category or starting with a specific letter in 60 s	Number of verbal category, verbal letter and total written words

WAIS-R DS, Weschler intelligence scale-revised DS; TMT-A, trail making test-part A; TMT-B, trail making test-part B; SCWT, Stroop colour word test; HVLT-R, Hopkins verbal learning test-revised; COWAT, controlled oral word association test.

### MRI data acquisition

MRI acquisition was performed using a 3 T Siemens Magnetom Skyra scanner with a 64-channel phased-array head coil at Austin Health. The study was part of a larger-scale multimodal MRI study (55, 56), including anatomical MRI scans (T1-weighted and T2 FLAIR), diffusion-weighted imaging (DWI), and resting-state fMRI. Our study focused on the anatomical T1-weighted MRI scans acquired using a three-dimensional magnetisation prepared rapid gradient echo (MPRAGE) with the following parameters: 1 mm isotropic voxel; repetition time (TR) = 2,300 ms; echo time (TE) = 2.98 ms; voxel size = 1.0 mm^3^; field of view (FOV) = 256 mm; flip angle = 9°; 192 slices; 1.0 mm slice thickness; 256 × 256 matrix; and acquisition time (TA) = 5′43″. The MRI scans were conducted at two timepoints (see procedure); however, one participant withdrew from the post-chemotherapy MRI scan due to distress related to disease progression.

### MRI data processing

We performed a quality assessment of the raw scans using an in-house protocol. Specifically, all T1-weighted images were visually inspected using Mango Viewer (Version 4.0.1) for motion-related artefacts, such as ghosting and striping, and other quality-related issues, such as signal inhomogeneity and susceptibility artefact. All scans passed the quality assessment.

Structural images were processed and analysed using the FreeSurfer image analysis suite Version 7.4.1 (http://surfer.nmr.mgh.harvard.edu). This software performs a series of automated steps, including: (1) skull stripping; (2) Talairach transformation; (3) segmentation of subcortical white matter and deep grey matter structures; (4) cortical surface reconstruction; (5) parcellation of the cerebral cortex based on the Desikan et al. (62); and (6) calculation of cortical thickness and surface area within these regions (63). Quality assurance of the registration and segmentation was undertaken by visual inspection using the ENIGMA protocol available at https://enigma.ini.usc.edu/protocols/imaging-protocols/. No participants were excluded from the analyses.

### Regions of interest

**Figure 1:**
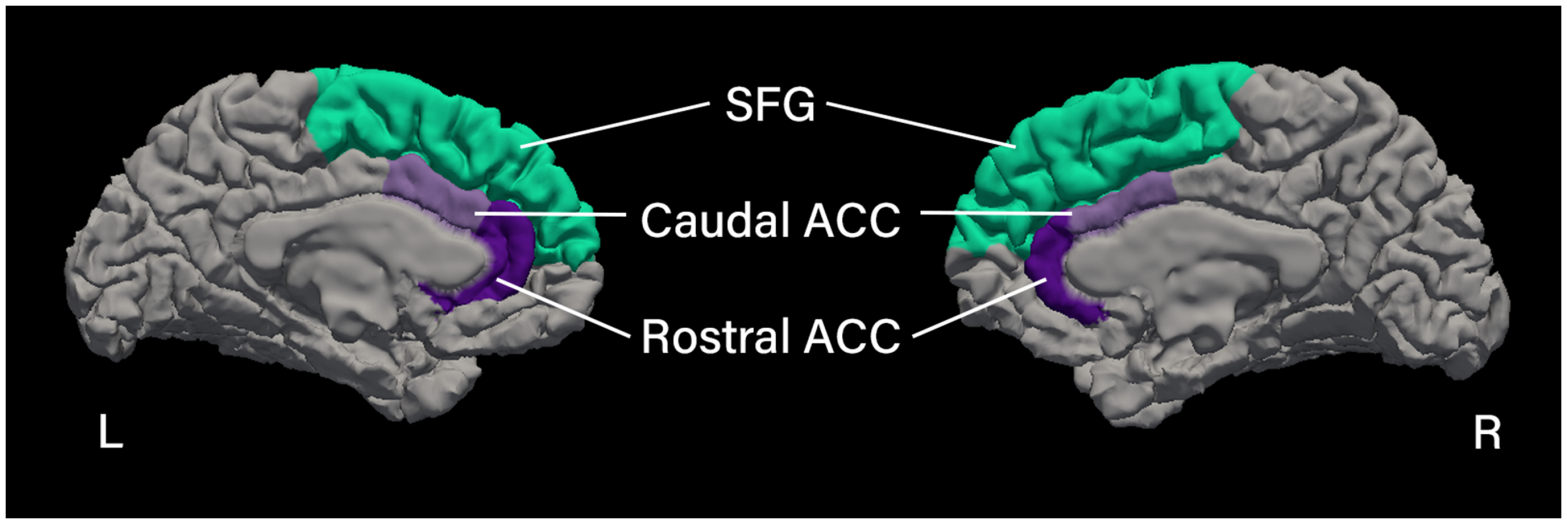
Cortical regions of interest. Medial view of the left and right hemispheres showing the cortical parcellation of the regions of interest based on the Desikan et al. (62), processed using FreeSurfer image analysis suite (Version 7.4.1; http://surfer.nmr.mgh.harvard.edu). SFG, superior frontal gyrus; ACC, anterior cingulate cortex.

Regions of interest (ROIs) were chosen based on prior literature, showing these cortical regions to be vulnerable in people with cancer (14, 20, 23, 24). Specifically, we selected the following six cortical regions from the Desikan et al. (62), the right and left caudal part of the ACC, the right and left rostral part of the ACC, and the right and left SFG (see Figure 1 for cortical ROIs). For each ROI, cortical thickness and surface area were calculated.

### Statistical analyses

For the normative analyses of the neuropsychological test scores (Aim 1), raw test scores were converted to *T-*scores using published normative data, adjusted for age, gender, and education. Participants were classified as “impaired” on a particular cognitive test if their *T*-score was ≤30, corresponding to 2 standard deviations below the normative mean (10).

For the normative analyses of brain morphology (Aim 2), we used the CentileBrain framework, developed by the ENIGMA Lifespan Group (49). CentileBrain is an open access web portal (https://centilebrain.org) that contains the normative models and an online tool for generating normative deviation scores for user-provided measures of subcortical volume and cortical thickness and surface. CentileBrain enables individualised evaluation of these neuroanatomical scores relative to age- and sex-specific reference distributions. The normative models were generated by applying Gaussian Process Regression to a normative reference population of 37,407 healthy controls encompassing a significant portion of the human lifespan (3–90 years) (49) to generate centile scores for key brain morphological metrics, allowing for the quantification of individual deviations of brain morphometry. The model parameters in the normative framework (CentileBrain) were applied to each Freesurfer derived regional cortical thickness and surface area measure of our regions of interest (caudal and rostral ACC, and SFG) from our patients with aggressive lymphoma. For each measure in each participant, we used CentileBrain to estimate the degree of normative deviation from the reference population mean as a *z*-score (computed by subtracting the estimated value from the raw value of that measure and then dividing the difference by the root mean square error of the model.) A positive or negative *z*-score indicates that the value of the corresponding morphometric measure is higher or lower, respectively, than the normative mean. Per previous literature (54), we defined regional *z*-scores as infranormal when below −1.96 or supranormal when above 1.96, corresponding to the 5th percentile and 95th percentile, respectively.

## Results

**Figure 2:**
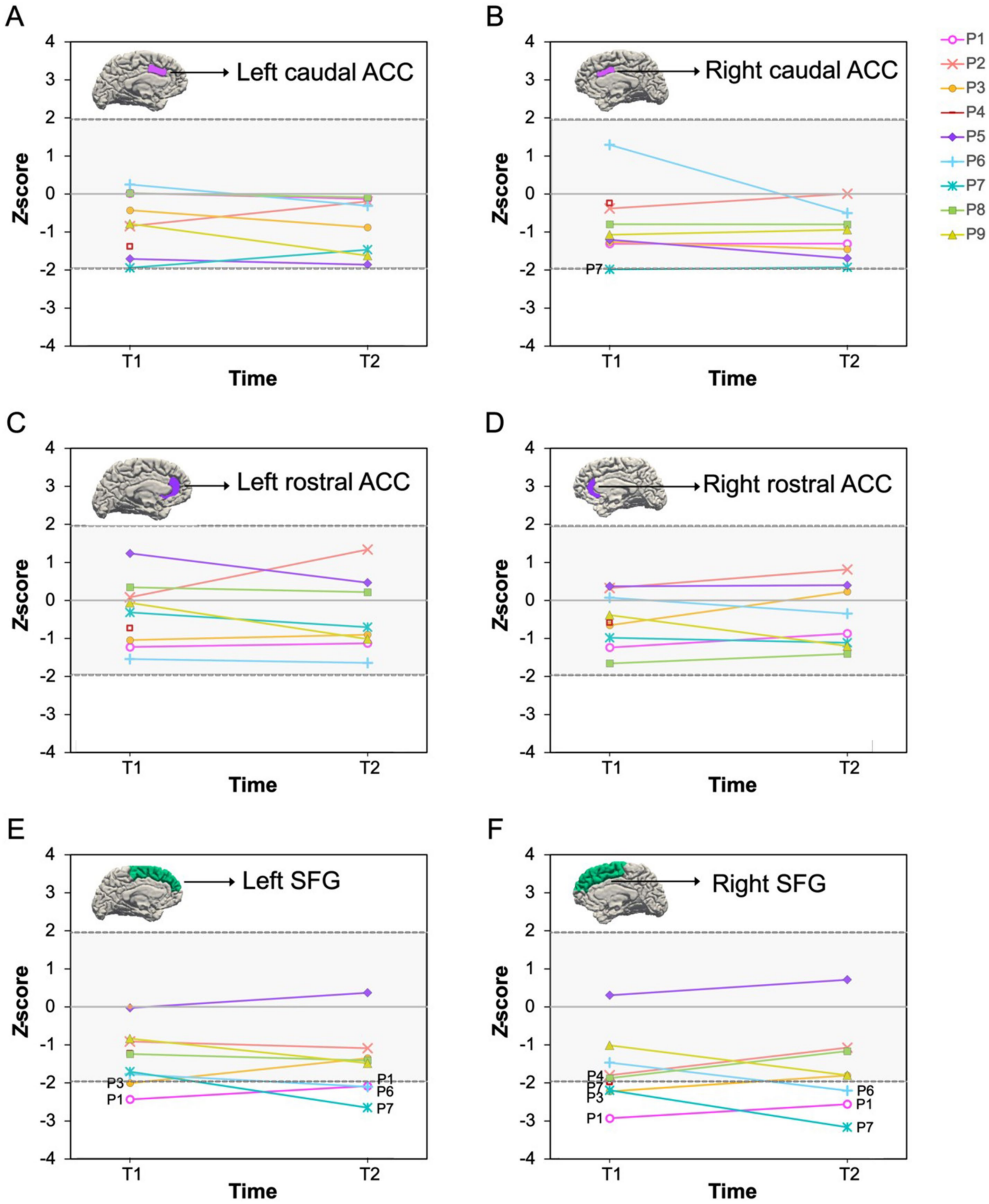
Cortical thickness *Z*-scores pre- and post-chemotherapy. P1–P9 represent participants 1–9. T1, pre-chemotherapy; T2, post-chemotherapy. The shaded area is the normal *Z*-score range (−1.96–1.96). **(A–F)** Represent scores for each ROI: **(A)** left caudal ACC; **(B)** right caudal ACC; **(C)** left rostral ACC; **(D)** right rostral ACC; **(E)** left SFG; **(F)** right SFG. ACC, anterior cingulate cortex; SFG, superior frontal gyrus.

**Figure 3:**
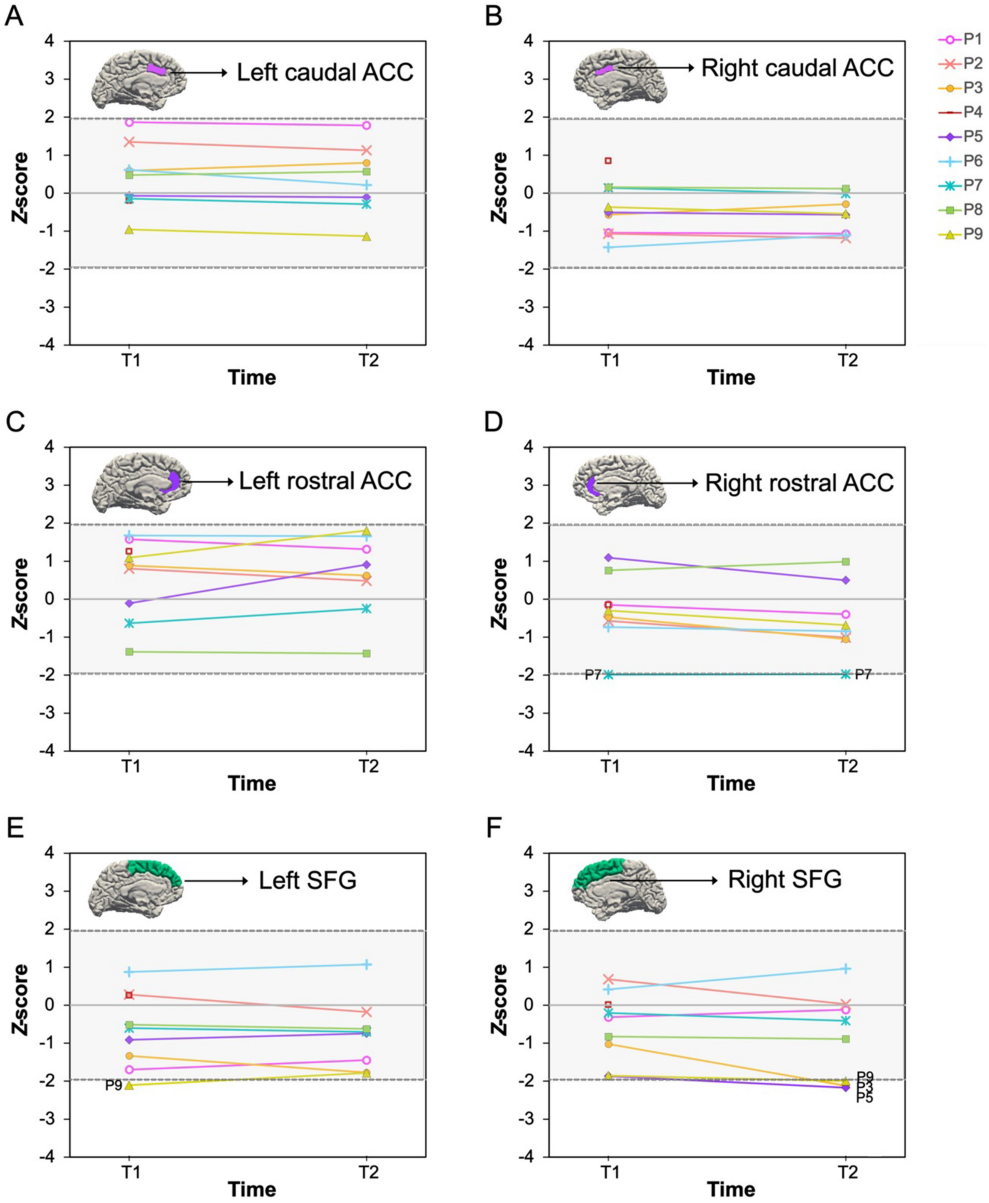
Cortical surface area *Z*-scores pre- and post-chemotherapy. P1–P9 represent participants 1–9. T1, pre-chemotherapy; T2, post-chemotherapy. The shaded area is the normal *Z*-score range (−1.96–1.96). **(A–F)** Represent scores for each ROI: **(A)** left caudal ACC; **(B)** right caudal ACC; **(C)** left rostral ACC; **(D)** right rostral ACC; **(E)** left SFG; **(F)** right SFG. ACC, anterior cingulate cortex; SFG, superior frontal gyrus.

**Figure 4:**
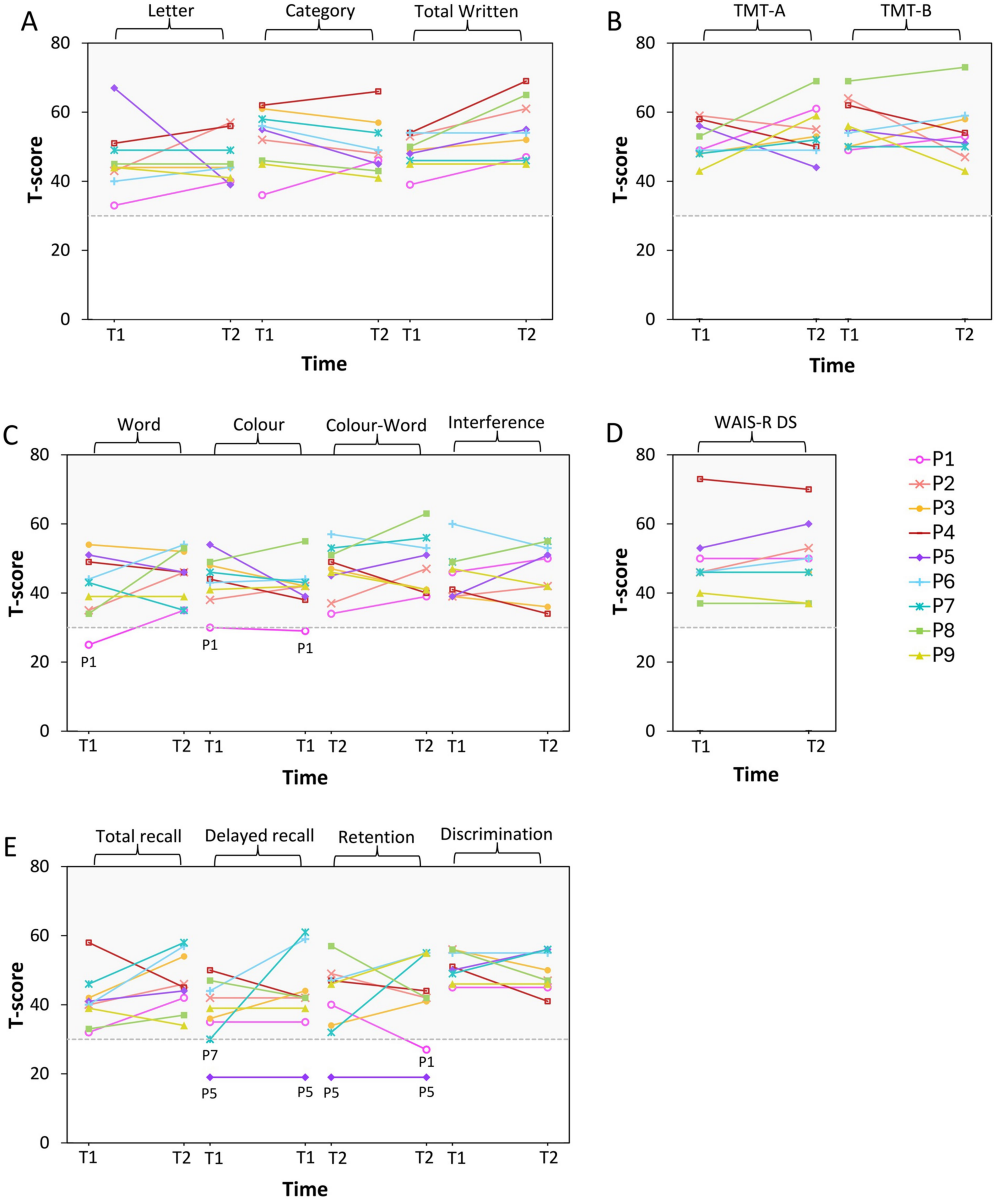
Neuropsychological test *T*-scores pre- and post-chemotherapy. P1–P9 represent participants 1–9. T1, pre-chemotherapy; T2, post-chemotherapy. The shaded area is the normal range (> 30). **(A–E)** Represent scores for each cognitive test: **(A)** COWAT; **(B)** TMT; **(C)** SCWT; **(D)** WAIS-R digit span; **(E)** HVLT-R.

As shown in Figures 2–4, we observed substantial between-subject variability in the results over time and across outcome measures in the nine participants. Impaired cognitive functioning was noted in the domains of executive functioning and verbal learning and memory. Infranormalities were more pronounced for cortical thickness than surface area and were more evident in the SFG compared to the ACC. Below, the results for each participant are presented as individual cases.

### Participant 1

A 47-year-old male diagnosed with stage 2A HL, treated with 6 cycles of the ABVD (adriamycin, bleomycin, vinblastine, and dacarbazine) chemotherapy regimen. Pre-chemotherapy, impaired cognitive function was observed in executive functioning (SCWT word, *T* = 25; colour, *T* = 30). Post-chemotherapy, impaired cognitive function persisted in executive functioning (SCWT colour, *T* = 29) and became evident in verbal learning and memory (HVLT retention, *T* = 27). Infranormal cortical thickness values in the left and right SFG were observed both pre-chemotherapy (*z* = −2.43, *p* = 0.015; *z* = −2.93, *p* = 0.003) and post-chemotherapy (*z* = −2.08, *p* = 0.038; *z* = −2.56, *p* = 0.010).

### Participant 2

A 66-year-old male diagnosed with stage 1A Grade 3B follicular lymphoma, treated with 6 cycles of the R-CHOP (rituximab, cyclophosphamide, doxorubicin, vincristine, and prednisolone) chemotherapy regimen. He scored within the normal range on all cognitive tests both pre- and post-chemotherapy. *Z*-scores of the six brain ROIs were also all within the normal range at both timepoints.

### Participant 3

A 69-year-old male diagnosed with stage 1A DLBCL, who underwent 7 cycles of the R-CHOP & 2 cycles of the high dose IT MTX (intrathecal methotrexate) chemotherapy regimen. He scored within the normal range on all cognitive tests at both timepoints. However, pre-chemotherapy, he exhibited infranormal cortical thickness values in the left and right SFG (*z* = −2.00, *p* = 0.046; *z* = −2.22, *p* = 0.026). Post chemotherapy, cortical thickness values of the bilateral SFG normalised and were within normal range. However, the surface area of the right SFG was identified as infranormal (*z* = −2.12, *p* = 0.034).

### Participant 4

A 78-year-old female diagnosed with stage 4A DLBCL, treated with 6 cycles of the Mini R-CHOP chemotherapy regimen. She scored within the normal range on all cognitive tests at both timepoints. However, pre-chemotherapy, cortical thickness of the right SFG was identified as infranormal (*z* = −1.98, *p* = 0.048). An MRI scan was not administered post-chemotherapy due to disease progression.

### Participant 5

A 72-year-old male diagnosed with stage 1A DLBCL, received 2 cycles of the R-CHOP chemotherapy regimen. He demonstrated impaired cognitive function in verbal learning and memory both pre-chemotherapy (HVLT delayed recall, *T* = 19; retention, *T* = 19) and post-chemotherapy (HVLT delayed recall, *T* = 19; retention, *T* = 19). Pre-chemotherapy, all brain regional z-scores were within the normal range. However, post-chemotherapy, surface area of the right SFG was identified as infranormal (*z* = −2.17, *p* = 0.030).

### Participant 6

A 29-year-old male diagnosed with stage 4B HL, who underwent 4 cycles of the Esc-BEACOPP (escalated BEACOPP: bleomycin, etoposide, doxorubicin, cyclophosphamide, vincristine, procarbazine, and prednisolone) chemotherapy regimen. He scored within the normal range on all cognitive tests at both timepoints. Pre-chemotherapy, *z*-scores of the six brain ROIs were also all within the normal range. However, infranormal cortical thickness values were evident in both the left and right SFG (*z* = −2.10, *p* = 0.036; *z* = −2.20, *p* = 0.028) post-chemotherapy.

### Participant 7

A 60-year-old male diagnosed with stage 2A DLBCL, treated with 4 cycles of the R-CHOP chemotherapy regimen. Pre-chemotherapy, he demonstrated impaired cognitive function in verbal learning and memory (HVLT delayed recall, *T* = 30). Post-chemotherapy, his verbal learning and memory function had normalised, and all cognitive test scores were within the normal range. Pre-chemotherapy, the cortical thickness of the right caudal ACC (*z* = −1.98, *p* = 0.048) and right SFG (*z* = −2.19, *p* = 0.029), as well as the surface area of the right rostral ACC, were identified as infranormal (*z* = −1.99, *p* = 0.047). Post-chemotherapy, the cortical thickness of the right caudal ACC normalised, however, the cortical thickness of the right SFG (*z* = −3.17, *p* = 0.002) and surface area of the right rostral ACC (*z* = −1.98, *p* = 0.048) remained infranormal. Additionally, the cortical thickness of the left SFG was identified as infranormal (*z* = −2.65, *p* = 0.008).

### Participant 8

A 63-year-old female diagnosed with stage 1A DLBCL, who received 3 cycles of the R-CHOP chemotherapy regimen. She scored within the normal range on all cognitive tests both pre- and post-chemotherapy. *Z*-scores of the six brain ROIs were also all within the normal range at both timepoints.

### Participant 9

A 56-year-old male diagnosed with stage 2B DLBCL, treated with 3 cycles of the R-CHOP chemotherapy regimen. He scored within the normal range on all cognitive tests at both timepoints. However, pre-chemotherapy, the surface area in the left SFG was identified as infranormal (*z* = −2.11, *p* = 0.035). Post-chemotherapy, values of the surface area of the left SFG normalised and were within normal range. However, the surface area of the right SFG was identified as infranormal (*z* = −1.99, *p* = 0.047).

## Discussion

Our novel study employed normative analysis to examine longitudinal changes in cognitive functioning and brain morphology in people with aggressive lymphoma. As hypothesised, impaired cognitive functioning and infranormal cortical thickness and surface area were observed, with considerable variability across participants and time points. Despite this heterogeneity, some commonalities were observed. Specifically, impaired cognitive functioning was observed in the domains of executive functioning and verbal learning and memory (in a small subset of patients). Additionally, infranormalities were more pronounced for cortical thickness than surface area, and more evident in the SFG compared to the ACC.

The finding that impaired cognitive functioning was mainly observed in tests of executive functioning and verbal learning and memory, contrasts with prior research on people with aggressive lymphoma, which have implicated a broader range of domains, including processing speed, attention/working memory, and verbal fluency (6, 8, 17). This inconsistency may be attributed to the stringent criteria we used to define cognitive impairment (ICCTF guidelines) resulting in mild impairments in other domains going undetected. Supporting this notion, a meta-analysis of 13 studies found that only executive function and memory (from seven cognitive domains in total) showed evidence of impaired functioning in cancer populations treated with chemotherapy (13). This suggests these areas may be more consistently impacted across studies, while other domains may exhibit more subtle changes.

In particular, verbal learning and memory may be vulnerable to CRCI, as suggested by our finding that these were the most frequently impaired cognitive domains in a subset of our patients (participants 1, 5, and 7). However, despite this small subgroup, this is supported by other studies of people with haematological malignancies (29, 64), which reported verbal learning and memory are especially prone to impairment both before and after treatment. For instance, Correa et al. found that people exhibited significantly poorer verbal memory performance compared to healthy controls both prior to and one year after undergoing hematopoietic stem cell transplantation (HSCT) – which involves a high-dose chemotherapy regimen – whereas other cognitive domains were only impacted at one year post-HSCT (29). Similarly, Syrjala et al. found that, while other cognitive functions like attention and processing speed showed signs of recovery, verbal recall remained persistently impaired from 80 days to five years post-HSCT (64). Collectively, this suggests that verbal learning and memory may be uniquely susceptible to impairment in this population due to the combined effects of the cancer and the cumulative impact of neurotoxic effects of chemotherapy.

Providing insight into the potential neural underpinnings of CRCI, we observed infranormalities in the SFG and ACC. This aligns with an extensive body of research demonstrating neuroanatomical alterations in frontal regions of non-CNS cancer populations (23, 24, 65). Nonetheless, while the majority of our participants showed infranormalities in the SFG, only one patient was found to exhibit infranormalities in the ACC (who also showed impaired cognitive function in verbal learning and memory). This is intriguing, particularly in light of the meta-analysis by Niu et al. (24) which found reductions in grey matter density in the right ACC and SFG among cancer populations treated with chemotherapy across all non-CNS cancer studies, whereas only the right SFG in their sub-analysis of breast cancer studies. This suggests alterations in frontal regions may vary, with the SFG being more consistently affected, while changes in the ACC may be more variable depending on cancer-type, patient characteristics or treatment factors.

Similarly, infranormalities were more pronounced for cortical thickness than surface area, suggesting the former may be more susceptible to chemotherapy or cancer-related factors. MRI research measuring cortical thickness and surface area is limited, with most studies focusing on grey matter volume or density. However, a recent study by Wu et al. assessed cortical thickness, surface area and volume in people with lung cancer both before and 2–4 months after chemotherapy (38). They observed significantly lower cortical thickness in the frontal, temporal, and parietal regions of participants pre-chemotherapy, whereas significant volume reductions were limited to the temporal regions, and no differences were found in surface area. Furthermore, only cortical thickness showed a significant decline post-chemotherapy, while surface area and volume remained unchanged (38). This suggests that grey matter volume changes noted in the broader literature may be largely attributable to cortical thickness rather than surface area. This is plausible given cortical thickness and surface area are influenced by different cellular processes and genetic origins (39, 40), and grey matter volume is the product of both measures (41). Thus, our findings extend prior research indicating that changes in grey matter volume may be largely driven by alterations in cortical thickness rather than surface area, underscoring the importance of examining these variables separately to understand the mechanisms involved in cancer and its treatment.

Despite some overlap in the type of impairments and infranormalities observed, there was large variability in cognitive functioning and neuroanatomical trajectories over time. Some participants exhibited cognitive impairments or infranormalities before chemotherapy which later normalised 6–8 weeks post chemotherapy, others showed impairments only after chemotherapy, and some displayed both. Prior research has similarly noted diversity in cognitive outcomes over time (66). For instance, Jansen et al. found that 33% of breast cancer participants exhibited significant cognitive decline following chemotherapy, while 17% showed notable improvements in cognitive functioning (66). Such patterns highlight the person-specific evolution of CRCI and related brain changes, which may be influenced by a complex interplay of factors, including pre-existing vulnerabilities, treatment effects, and recovery mechanisms.

Clinically, the heterogeneity found across participants and time underscores the importance of individualised approaches or normative analyses in managing CRCI. Unlike group-level analyses, which may overlook between-subject variability, normative analysis allows for the precise characterisation of cognitive and neuroanatomical changes at the individual level, capturing the unique trajectories of decline and recovery people may experience (20). This is crucial in the context of CRCI, given the high variability in impairments and neuroanatomical changes observed in our study; some participants exhibited deficits in specific domains, such as executive function or verbal memory, while others did not show any impairment. Using these individualised profiles, clinicians can develop interventions specifically designed to address each person’s unique needs. Indeed, evidence has shown that group-based programs, such as a cognitive rehabilitation program (67), and a mindfulness-based stress reduction program (68), can significantly improve cognitive functioning in cancer survivors who have undergone chemotherapy. Our previous qualitative study in the same population, explored participants’ experience with comprehensive cognitive assessments at a time of heightened stress related to a new diagnosis of aggressive lymphoma and rapid commencement of treatment. The study identified the following were important to participants: demands of data collection were minimised; a clinician from the service was included; the tasks were seen as inherently interesting; and care was taken to provide empathic support throughout (69). Our results suggest tailoring such programs to individual needs may improve outcomes further. Furthermore, our study demonstrates the value of utilising CentileBrain when assessing people with cancer. The program provides clinicians with information on any structural brain changes at the level of the individual. This information could be helpful in early identification of those at higher risk of CRCI, enabling more timely and tailored interventions to mitigate or alleviate cognitive decline (70).

Nonetheless, there are several limitations in this study that should be addressed. This study included a small number of people with aggressive lymphoma. It is important to point out that the aim of this study was to perform an individualised normative analysis to assesses how far each individual patient deviates from the norm. Moreover, the large reference cohort sample of 37,407 healthy individuals, allowed us to identify unique patterns of deviations reflecting potentially clinically relevant changes. Our results provide support for a normative analysis to be used in future studies using aggressive lymphoma samples that are large enough to capture the pathological variability in brain morphology. Second, the follow-up period was limited to approximately 6–8 weeks after chemotherapy (with some variability in the timing of post-treatment assessments). This time period may have missed the detection of longer-term cognitive or neuroanatomical alterations that could develop or resolve. This is particularly relevant given that grey matter volume in the SFG has been shown to be positively associated with time since chemotherapy, suggesting this region may gradually recover (24). Furthermore, McDonald et al. reported that while some brain regions showed signs of recovery between one month and one year post-chemotherapy, other regions did not, highlighting the variability in recovery across different brain areas (71). Similarly, cognitive functions like verbal recall and executive functioning have been reported to remain impaired up to five years post-treatment in cancer survivors (64, 72). Therefore, future research should extend follow-up to at least one-year post-chemotherapy, to better capture the long-term cognitive and neuroanatomical changes in survivors of aggressive lymphoma, given their increasing long-term survivorship. Nonetheless, this study is unique in obtaining pre-cancer treatment data in this under-researched cancer population, offering critical insights into the early effects of aggressive lymphoma and its treatment.

A third limitation of this study was the reliance on objective measures of cognitive function (10). Subjective reports, such as patient-reported cognitive concerns (e.g., the Functional Assessment of Cancer Therapy-Cognitive Function [FACT-Cog] scale) (73) may provide more sensitive and early indicators of cognitive changes. This is especially relevant for high-functioning individuals who often report cognitive concerns before objective measures detect a decline in cognitive functioning (74). Thus, future research should incorporate both objective and subjective assessments to sufficiently capture the complexities of patient experiences beyond what objective tests alone can reveal (10).

Finally, because of our strong, anatomical, a-priori hypothesis, our neuroimaging analysis was restricted to specific regions of interest thereby potentially neglecting other brain regions that may be affected by cancer and its treatment. This is an important limitation because research has shown cognitive changes in cancer populations are not confined to these (frontal) regions alone. For example, Li and Caeyenberghs found reduced grey matter density in other areas of the frontal lobe, such as the middle frontal gyrus, as well as in temporal and parietal regions, suggesting these areas may also play a role in CRCI (14). Future research should therefore include a range of brain regions to provide a more comprehensive understanding of the neural underpinnings of CRCI.

## Conclusion

This study represents a significant advancement in understanding CRCI in people with aggressive lymphoma by using normative analysis to explore cognitive function and neuroanatomical changes from pre- to post-chemotherapy. Unlike traditional group-based studies, this method allowed for the detailed examination of individual variability, revealing diverse trajectories of cognitive impairment and brain structural alterations that may otherwise be missed. The use of normative data from CentileBrain further enhanced the sensitivity of detecting subtle changes in cortical thickness and surface area, offering a more biological-specific understanding of the neural underpinnings of CRCI. The study’s focus on both cortical thickness and surface area is particularly novel, highlighting the differential impacts of chemotherapy on these measures and underscoring the need to consider both in future research. Moreover, the inclusion of pre-treatment data provides a deeper understanding of the early neuroanatomical and cognitive changes that may occur for this population. Collectively, these insights provide a valuable foundation for developing more personalised interventions tailored to the specific cognitive and neural profiles of lymphoma survivors, paving the way for improved clinical care to alleviate and mitigate the impact of CRCI on long-term quality of life.

## Data Availability

The raw data supporting the conclusions of this article will be made available by the authors, without undue reservation.
